# Clinical research of Olanzapine for prevention of chemotherapy-induced nausea and vomiting

**DOI:** 10.1186/1756-9966-28-131

**Published:** 2009-09-23

**Authors:** Lijun Tan, Jiangtao Liu, Xiuli Liu, Jie Chen, Zhijun Yan, Huifen Yang, Daxin Zhang

**Affiliations:** 1 First Department of Oncology, the First Affiliated Hospital of Harbin Medical University, Heilongjiang, 150001, China; 2 Department of Otolaryngology, the First Affiliated Hospital of Harbin Medical University, Heilongjiang, 150001, China

## Abstract

**Background:**

This study was designed to mainly evaluate the activity and safety of olanzapine compared with 5-hydroxytryptamine3(5-HT_3_) receptor antagonists for prevention of chemotherapy-induced nausea and vomiting(CINV) in patients receiving highly or moderately emetogenic chemotherapy (HEC or MEC). The second goal was to evaluate the impact of olanzapine on quality of life (QoL) of cancer patients during the period of chemotherapy.

**Methods:**

229 patients receiving highly or moderately emetogenic chemotherapy were randomly assigned to the test group [olanzapine(O) 10 mg p.o. plus azasetron (A) 10 mg i.v. and dexamethasone (D) 10 mg i.v. on day 1; O 10 mg once a day on days 2-5] or the control group (A 10 mg i.v. and D 10 mg i.v. on day 1; D 10 mg i.v. once a day on days 2-5). All the patients filled the observation table of CINV once a day on days 1-5, patients were instructed to fill the EORTC QLQ-C30 QoL observation table on day 0 and day 6. The primary endpoint was the complete response (CR) (without nausea and vomiting, no rescue therapy) for the acute period (24 h postchemotherapy), delayed period (days 2-5 poschemotherapy), the whole period (days 1-5 postchemotherapy). The second endpoint was QoL during chemotherapy administration, drug safety and toxicity.

**Results:**

229 patients were evaluable for efficacy. Compared with control group, complete response for acute nausea and vomiting in test group had no difference (p > 0.05), complete response for delayed nausea and vomiting in patients with highly emetogenic chemotherapy respectively improved 39.21% (69.64% versus 30.43%, p < 0.05), 22.05% (78.57% versus 56.52%, p < 0.05), complete response for delayed nausea and vomiting in patients with moderately emetogenic chemotherapy respectively improved 25.01% (83.07% versus 58.06%, p < 0.05), 13.43% (89.23% versus 75.80%, p < 0.05), complete response for the whole period of nausea and vomiting in patients with highly emetogenic chemotherapy respectively improved 41.38% (69.64% versus 28.26%, p < 0.05), 22.05% (78.57% versus 56.52%, p < 0.05), complete response for the whole period of nausea and vomiting in patients with moderately emetogenic chemotherapy respectively improved 26.62% (83.07% versus 56.45%, p < 0.05), 13.43% (89.23% versus 75.80%, p < 0.05). 214 of 299 patients were evaluable for QoL. Comparing test group with control group in QoL evolution, significant differences were seen in global health status, emotional functioning, social functioning, fatigue, nausea and vomiting, insomnia and appetite loss evolution in favour of the test group (p < 0.01). Both treatments were well tolerated.

**Conclusion:**

Olanzapine can improve the complete response of delayed nausea and vomiting in patients receiving the highly or moderately emetogenic chemotherapy comparing with the standard therapy of antiemesis, as well as improve the QoL of the cancer patients during chemotherapy administration. Olanzapine is a safe and efficient drug for prevention of CINV.

## Background

Chemotherapy-induced nausea and vomiting is a significant side effect of cancer therapy for many years[1]. CR for acute period and delayed period in the patients receiving highly and moderately emetogenic chemotherapy with the use of 5-HT_3 _receptor antagonists plus dexamethasone is respectively 68%-90% and 47%-56%. Despite the use of 5-HT_3 _receptor antagonists plus dexamethasone has significantly improved the control of the acute CINV, the complete response for the delayed nausea and vomiting has not significantly improved comparing with the sole use of dexamethasone[2]. Recent studies have demonstrated additional improvement in the control of acute and delayed CINV with the use of two new agents, aprepitant, the first agent available in the new drug class of neruokinin-1 receptor antagonists, and palonosetron, a second-generation 5-HT_3 _receptor antagonist [3-5]. Because without of the application of the two new drugs in China, we still have many chance for improvement with the addition or substitution of new agents in current antiemetic regimens.

Olanzapine, an atypical antipsychotic drug, blocks multiple neurotransmitters: serotonin, at 5H2a, 5H2c, 5H3, and 5HT6 receptors, dopamine at D1, D2, D3 and D4 brain receptor, catecholamines at alpha 1 adrenergic receptors, acetylcholine at muscarinic receptors, and histamine at H1 receptors. Just for its action at multiple receptors sites, particularly at the D2 and 5H_3 _receptors, which appear to be involved in nausea and vomiting, suggest that it has potential antiemetic properties.

At first some case reports shew that olanzapine was effective in reduction nausea in advanced cancer patients with opioid-induced nausea [6,7]. Another study reported that olanzapine may decrease delayed emesis in 28 cancer patients treated with highly or moderately emetogenic chemotherapy [8]. Then a phase I study made sure the maximum tolerated dose of olanzapine which is 5 mg per day for the 2 days prior to chemotherapy and 10 mg per day for 7 days postchemotherapy[9]. It had safe and effective use for the prevention of delayed emesis in cancer patients receiving moderately to highly emetogenic chemotherapy such as cyclophosphamide, doxorubicin, cisplatin, and/or irinotecan. In a II stage trial of olanzapine[10] in combination with granisetron and dexamethasone for prevention of CINV, the combination therapy proved to be highly effective in controlling acute and delayed CINV in patients receiving highly and moderately emetogenic chemotherapy. CR for acute period, delayed period in ten patients receiving highly emetogenic chemotherapy is respectively 100% and 80%. Results for moderately emetogenic chemotherapy were similar. In order to reduce the side effect of dexamethasone, Navari designed a II stage trial to determine the control of acute and delayed CINV in patients receiving moderately and highly emetogenic chemotherapy with the combined use of palonosetron, olanzapine and dexamehthasone which was given on day 1 only. For the first cycle of chemotherapy, the complete response (no emesis, no rescue) for the acute, delayed and overall period was respectively 100%, 75%, and 75% in 8 patients receiving HEC and 97%, 75%, and 72% in 32 patients receiving MEC. Patients with no nausea for the acute, delayed, and overall period was respectively 100%, 50% and 50% in 8 patients receiving HEC and was 100%,78%, and 78% in 32 patients receiving MEC. The result shew that olanzapine combined with a single dose of dexamethasone and a single dose of palonosetron was very effective in controlling acute and delayed CINV in patients receiving both HEC and MEC.

Based on these data, olanzapine appear to be a safe and effective agent for prevention acute and delayed CINV in spite of a few of patients. At present the antiemetic regimen is the combination of 5-HT_3 _receptor antagonist, dexamethasone and/or metoclopramide, diazepam in China. In an attempt to improve the complete remission of the acute and delayed emesis, we preformed a study used with the combination of olanzapine, azasetron and dexamethasone for prevention acute and delayed nausea and vomiting induced by highly or moderately emetogenic chemotherapy.

## Methods

### Patient selection

The adult patients with a pathological diagnosis of malignant disease or previously treated by chemotherapy were enrolled onto the study and received either moderately emetogenic (oxaliplatin, carboplatin, epirubicin, adriamycin) or highly emetogenic (cisplatin, dacarbazine) chemotherapy. Patients were required to have adequate bone marrow (absolute neutrophil count ≥ 1,500/ul, HB ≥ 10 g/L, platelet count ≥ 80,000/ul), renal (serum creatinine ≤ 1.5 mg/dl) and liver (serum bilirubin ≤ 1.5 mg/dl) functions, normal cardiac function, ECOG performance status ≤ 2, no nausea in the 24 h prior to beginning olanzapine or chemotherapy, no severe cognitive compromise, no known history of CNS disease (e.g., uncontrolled brain metastases, seizure disorder), no antipsychotic disease, no concurrent abdominal radiotherapy, no know hypersensitivity to olanzapine, no history of uncontrolled diabetes mellitus, no concurrent medical disease. All patients gave written informed consent to participate in the trial.

### Study design and antiemetic treatment

All eligible patients were randomized into test group and control group according to the random digits table. On the day of chemotherapy, day 1, the test group patients received the antiemetic regimen consist of olanzapine 10 mg p.o., azasetron 10 mg, i.v. and dexamethasone 10 mg i.v., the control group patients received a standard pre-treatment antiemetic regimen consist of azasetron 10 mg, i.v. and dexamethasone 10 mg, i.v. Day 2-5, the test group patients received olanzapine 10 mg p.o., the control group patients received dexamethasone 10 mg, i.v.. Patients were permitted to take other antiemetic therapy for nausea and/or emesis based on clinical circumstances.

### Study endpoints

The primary endpoint was CR, the second endpoint was QoL, drug safety and toxicity. CINV was graded by CTCAE V 3.0, QoL was evaluated according to EORTC QLQ-C30.

### Assessment procedures

All of the enrolled patients whose data such as age, sex, height, weight should be recorded underwent a complete physical examination, laboratory assessment (i.e. blood analysis, liver function, renal function, blood glucose, blood lipids) before chemotherapy. At days 1-5 postchemotherapy patients used the observation table of CINV to record the response of the patients (mainly recorded the degree of CINV, as well as whether to take the remedial treatment to relieve nausea and vomiting), at same time patients were instructed to fill the EORTC QLQ-C30 QoL observation table on day 0 and day 6.

### Statistical analyses

Statistical analyses were carried out using SPSS14.0. The percentage of patients with complete response for acute period, delayed period and the overall period (0-120 h postchemotherapy) was calculated separately in test group and control group, as well as every level of nausea and vomiting. The X^2 ^test was utilized to analyze complete response. The Wilcoxon-signed rank test was used to compare QoL data before and after chemotherapy. Student's t-test was used to compare parametric QoL data postchemotherapy between groups. The Mann-Whitney U test was performed to compare non-parametric QoL data postchemotherapy between groups. P-value of < 0.05 was considered as statistically significant.

## Results

### Patients characteristics

From January 2008 to August 2008,229 patients were randomly enrolled onto the study. All patients were evaluable for efficacy and toxicity. Groups were comparable regarding age, sex and drug which distribution were balanced (p > 0.05) (Table 1). All patients received chemotherapy. There were 108 patients in test group and 106 patients in control group who took part in filling QoL assessment.

**Table 1:** characteristics of patients in two groups

	**Test group**	**Control group**
Number of patients	121	108
Age range (mean standard deviation)		
male	40-73(54 ± 9.23)	41-74(54.5 ± 10.33)
female	27-68(48.25 ± 12.70)	18-67(49.58 ± 12.12)
Gender		
Male	72 (59.50%)	65 (60.20%)
Female	49 (40.50%)	43 (39.80%)
Drug		
Cisplain(75 mg/m^2^)	56 (46.30%)	44 (40.70%)
Oxaliplatin(85 mg/m^2^)	27 (22.30%)	26 (24.10%)
Epirubicin(90 mg/m^2^)	19 (15.7%)	22 (20.4%)
Carboplatin(AUC 5)	9 (7.40%)	4 (3.7%)
Adriamycin(50 mg/m^2^)	10 (8.3%)	10 (9.3%)
Dacarbazine(200 mg/m^2^)	0	2(1.9%)
Cancer type		
Lung	39	15
Stomach	9	12
Breast	23	31
Ovarian	10	2
Lymphoma	12	10
Oesophageal	5	6
Colorectal	16	14
Oropharyngeal	3	0
Teratoma	4	0
Gingival	0	3
Thymus	0	4
Cervical	0	4
Laryngeal	0	2
Malignant melanoma	0	3
Glioblastoma	0	2

### Primary efficacy analysis

Both of test group and control group had showed better efficacy on controlling CINV. Comparison of drug efficacy was shown in Table 2. Compared with control group, complete response for acute period in test group with highly or moderately emetogenic chemotherapy had no difference (p > 0.05), complete response for delayed nausea and vomiting in patients with highly emetogenic chemotherapy respectively improved 39.21%(69.64% versus 30.43%, p < 0.05), 22.05% (78.57% versus 56.52%, p < 0.05), complete response for delayed nausea and vomiting in patients with moderately emetogenic chemotherapy respectively improved 25.01%(83.07% versus 58.06%, p < 0.05), 13.43% (89.23% versus75.80%, p < 0.05), complete response for the whole period of nausea and vomiting in patients with highly emetogenic chemotherapy respectively improved 41.38% (69.64% versus 28.26%, p < 0.05), 22.05% (78.57% versus 56.52%, p < 0.05), complete response for the whole period of nausea and vomiting in patients with moderately emetogenic chemotherapy respectively improved 26.62% (83.07% versus 56.45%, p < 0.05), 13.43% (89.23% versus 75.80%, p < 0.05). Age was significantly correlated with acute, delayed and the whole period nausea in the level of 0.01.

**Table 2:** Complete response of CINV

	**Complete response (%)**											
	
	**AN**		**AV**		**DN**		**DV**		**NC**		**VC**	
						
	**H**	**M**	**H**	**M**	**H**	**M**	**H**	**M**	**H**	**M**	**H**	**M**
TG	94.64	98.46	91.07	96.92	69.64	83.07	78.57	89.23	69.64	83.07	78.57	89.23
CG	86.96	93.54	89.13	96.77	30.43	58.06	56.52	75.80	28.26	56.45	56.52	75.80
						
P value	> 0.05		> 0.05		< 0.05		< 0.05		< 0.05		< 0.05	

AN: acute nausea, AV: acute vomiting, DN: delayed nausea, DV: delayed vomiting, NC: nausea of whole period of chemotherapy, VC: vomiting of whole period of chemotherapy, TG: test group, CG: control group, H: highly emetogenic chemotherapy, M: moderately emetogenic chemotherapy

The results of further classification for CINV were shown in Table 3. Test group and control group had achieved better efficacy without of acute nausea and vomiting prior to level 3 and delayed acute nausea and vomiting prior to level 4. Complete response for level 1 acute nausea, level 3 delayed nausea and vomiting were 100% in test group, but there were no statistically difference compared with control group (p > 0.05). The efficacy for level 2 acute or delayed nausea and vomiting in test group were superior to control group (p < 0.05).

**Table 3:** Complete response of CINV in different grade

	**Complete response (%)**									
	
	**AN**		**AV**		**DN**			**DV**		
				
	**L1**	**L2**	**L1**	**L2**	**L1**	**L2**	**L3**	**L1**	**L2**	**L3**
TG	96.70	97.52	97.52	99.17	90.08	94.21	100	93.39	96.70	100
CG	100	87.04	97.22	91.66	82.40	62.96	99.07	89.81	76.85	99.07
P value	> 0.05	< 0.05	> 0.05	< 0.05	> 0.05	< 0.05	> 0.05	> 0.05	< 0.05	> 0.05

Definition of nausea according to CTCAE V 3.0

L1: Loss of appetite without alteration in eating habits

L2: Oral intake decreased without significant weight loss, dehydration or malnutrition; IV fluids, indicated < 24 hrs.

L3: inadequate oral caloric and/or fluid intake, IV fluids, tube feedings, or TPN indicated ≥ 24 hrs

L4: Life-threatening consequences

L5: Death

Definition of nausea according to CTCAE V 3.0

L1: 1 episode in 24 hrs

L2: 2-5 episodes in 24 hrs; IV fluids indicated < 24 hrs

L3: > = 6 episodes in 24 hrs; IV fluids, or TPN indicated > = 24 hrs

L4: Life-threatening consequences

L5: Death

### Secondary efficacy parameters

There were 214 patients whose QoL data could be evaluated. The QLQ-C30 responses were scored and analyzed according to algorithms in a scoring manual supplied by the EORTC Study Group on Quality of life. An increased score for a functional domain and global QoL scale represents an improvement of functioning, an decreased score for a symptom scale represents an improvement of symptomatic problem. After chemotherapy an improvement in global health status, emotional functioning, cognitive functioning, pain, dyspnoea, insomnia, appetite loss were seen in test group, but no difference in cognitive functioning, dyspnoea and appetite loss were seen (p > 0.05). After chemotherapy an improvement in pain and dyspnoea were seen in the control group, but no difference in pain was seen (p > 0.05). Comparing test group and control group in QoL evolution, significant differences were seen in global health status, emotional functioning, social functioning, fatigue, nausea and vomiting, insomnia and appetite loss evolution in favour of test group (p < 0.01).

All the enrolled patients had completed the study. 73% of patients in test group had sleepiness during chemotherapy, but after chemotherapy weight, blood lipoid and blood glucose did not change significantly. Fatigue, headache, dry mouth, diarrhea were common adverse events in two groups but did not result in level 3 or 4 toxicity, which could be tolerated by two groups patients. Most patients in control group had disturbed sleep during chemotherapy which could be relieved by oral estazolam.

## Discussion

Although 5-HT_3 _receptor antagonists have been particularly effectively for the acute CINV [11-13], they have not effective against the delayed CINV in patients receiving highly or moderately emetogenic chemotherapy [14]. They have the same efficacy as dexamethation for prevention of the delayed CINV [2], so this study compared olanzapine regimen with the standard therapy regimen to evaluate their effect for CINV in patients receiving highly or moderately emetogenic chemotherapy. In the present study, the effect of two regimens were similar to the acute nausea and vomiting, but the olanzapine regimen protected more than two-thirds of patients from emesis after they received highly or moderately emetogenic chemotherapy and enabled them to avoid the use of rescue therapy during 2-4 days after chemotherapy, whereas treatment of control group with the currently available standard therapy protected approximately half of patients. The superiority of olanzapine for control of delayed nausea and vomiting caused by highly emetogenic chemotherapy is more than its roles on delayed nausea and vomiting caused by moderately emetogenic chemotherapy. In the assessments of complete response over the period after chemotherapy, the olanzapine regimen provided a substantial improvement of 41 and 26 percent points and 22 and 13 percent points over standard therapy in the prevention of nausea and vomiting after highly and moderately emetogenic chemotherapy, this represented a clearly meaningful benefit.

Recent studies demonstrated that the acute emesis is mainly associated with serotonin, so 5-HT_3 _receptor antagonists have a dramatically effect on the acute emesis in many trials, but delayed emesis seems to differ in its pathogenic mechanism from acute emesis because drugs that are so effective in preventing the acute emesis are less effective in the delayed period such as 5-HT_3 _receptor antagonist. Olanzapine blocks multiple neurotransmitters which are known mediators of CINV. Olanzapine appears to have activity in control acute and delayed nausea and vomiting.

According to CTCAE V3.0, level 1 of nausea means loss of appetite without alteration in eating habits, level 2 means oral intake decreased without significant weight loss, dehydration or malnutrition; IV fluids, indicated < 24 hrs, level 3 means inadequate oral caloric and/or fluid intake, IV fluids, tube feedings, or TPN indicated > = 24 hrs. Level 1 of vomiting means 1 episode in 24 hrs, level 2 means 2-5 episodes in 24 hrs; IV fluids indicated < 24 hrs, level 3 means > = 6 episodes in 24 hrs; IV fluids, or TPN indicated > = 24 hrs. This study of further classification for nausea and vomiting showed that olanzapine regimen had statistical difference in controlling level 2 acute or delayed nausea and vomiting from standard therapy regimen for CINV opposite to controlling level 1 nausea and vomiting. This meant that olanzapine could relieve the degree of acute or delayed nausea and vomiting and improve the efficacy of its antiemetic role.

Dexamethasone is effective as monotherapy and in combination with 5-HT_3 _receptor antagonist to prevent acute and delayed nausea and vomiting in patients receiving a chemotherapeutic regimens used for treatment of different cancers. However, one must be aware of potential toxic effects of dexamethasone. In a recent survey, moderate-to-severe side-effects noted for patients receiving dexamethasone for prophylaxis against delayed CINV included insomnia (45%), gastrointestinal symptoms (27%), agitation (25%), increased appetite (18%), weight gain (17%), rash (15%), depression on cessation of treatment (7%), hiccups (7%) and oral candidiasis (3%)[15]. In order to try one' best to relieve the side-effects of dexamethasone, olanzapine was separately used to prevent the delayed nausea and vomiting comparing with dexamethasone for delayed nausea and vomiting in patients receiving highly or moderately chemotherapy in this study. Olanzapine in combination with 5-HT_3 _receptor antagonist and dexamethasone was shown to be superior to 5-HT_3 _receptor antagonist and dexamethasone in controlling the acute and delayed CINV in patients receiving highly or moderately emetogenic chemotherapy, specifically for the delayed nausea and vomiting. The severe toxic effects of olanzapine was not seen in this clinical study. The most frequent side-effect was sleepiness which could effectively relieve insomnia and agitation caused by dexamethasone.

The diagnosis of cancer is a life-altering experience for anyone. Some cancer patients could have inevitable emotions that can interfere with medical care, family, diet, sleep, exercise. The more common diagnosed psychiatric conditions are depression, anxiety, adjustment disorders, delirium. Often, patients have mixed states or combinations of symptoms, such as depression and anxiety. Olanzapine is an atypical antipsychotic drug, some studies have demonstrated the antidepressant efficacy of olanzapine [16,17]. In this study, whether the use of olanzapine for five days could result in the improvement of QoL because of its antipsychotic effects, which need to further study for no relevant studies to be reported. But we observed olanzapine not only elevated the complete response for CINV, specially for the delayed nausea and vomiting but also improved the emotion, sleep, appetite of the cancer patients compared with the standard therapy regimen of antiemesis. Improvement of the cancer patients QoL during chemotherapy can make the patients more confidence for treatment which can make the patients complete the whole treatment. This will result in the improvement of the clinical efficacy.

## Conclusion

In summary, this study demonstrated that olanzapine has obtained the better efficacy on being safely used for preventing the CINV. Olanzapine can improve the complete response of delayed nausea and vomiting in patients receiving the highly or moderately emetogenic chemotherapy comparing with the standard therapy of antiemesis, as well as improve the QoL of the cancer patients during chemotherapy. Olanzapine is a safe and efficient drug for prevention of CINV. Further study should be done to compare the efficacy of olanzapine with aprepitant or palonosetron on prevention of CINV through large sample study.

## Competing interests

The authors declare that they have no competing interests.

## Authors' contributions

LT designed and carried out this study, drafted the manuscript. DZ conceived of the study, JL participated in its design and modified the manuscript. XL, JC, ZY and HY provided the patients for study. JP, JL and YR helped with the clinical observation. All authors read and approved the final manuscript.
